# Emerging orthobunyaviruses associated with CNS disease

**DOI:** 10.1371/journal.pntd.0008856

**Published:** 2020-10-28

**Authors:** Arthur Wouter Dante Edridge, Lia van der Hoek

**Affiliations:** 1 Laboratory of Experimental Virology, Department of Medical Microbiology and Infection Prevention, Amsterdam institute for Infection & Immunity, Amsterdam UMC, University of Amsterdam, the Netherlands; 2 Global Child Health Group, Emma Children’s Hospital, Amsterdam UMC, University of Amsterdam, the Netherlands; University of Liverpool, UNITED KINGDOM

## Abstract

The *Orthobunyavirus* genus comprises a wide range of arthropod-borne viruses which are prevalent worldwide and commonly associated with central nervous system (CNS) disease in humans and other vertebrates. Several orthobunyaviruses have recently emerged and increasingly more will likely do so in the future. Despite this large number, an overview of these viruses is currently lacking, making it challenging to determine importance from a One Health perspective. Causality is a key feature of determining importance, yet classical tools are unfit to evaluate the causality of orthobunyaviral CNS disease. Therefore, we aimed to provide an overview of orthobunyaviral CNS disease in vertebrates and objectify the causality strength of each virus. In total, we identified 27 orthobunyaviruses described in literature to be associated with CNS disease. Ten were associated with disease in multiple host species of which seven included humans. Seven viruses were associated with both congenital and postnatal CNS disease. CNS disease-associated orthobunyaviruses were spread across all known *Orthobunyavirus* serogroups by phylogenetic analyses. Taken together, these results indicate that orthobunyaviruses may have a common tendency to infect the CNS of vertebrates. Next, we developed six tailor-made causality indicators and evaluated the causality strength of each of the identified orthobunyaviruses. Nine viruses had a ‘strong’ causality score and were deemed causal. Eight had a ‘moderate’ and ten a ‘weak’ causality score. Notably, there was a lack of case-control studies, which was only available for one virus. We, therefore, stress the importance of proper case-control studies as a fundamental aspect of proving causality. This comprehensible overview can be used to identify orthobunyaviruses which may be considered causal, reveal research gaps for viruses with moderate to low causality scores, and provide a framework to evaluate the causality of orthobunyaviruses that may newly emerge in the future.

## Author summary

Orthobunyaviruses are commonly implicated as causes of CNS disease. Well known examples include La Crosse virus, the primary cause of encephalitis in children in the United States, and Schmallenberg virus, responsible for causing large outbreaks of CNS defects in newborn ruminants in Europe and Asia. In addition, several other less-known and novel orthobunyaviruses have also been implicated in CNS disease, yet association does not imply causality. Considering that nearly one hundred orthobunyaviruses are known to date and more are discovered every year, it is important to determine which orthobunyaviruses are relevant in the context of CNS disease, yet an overview of such information was lacking. To solve this problem, we first identified all orthobunyaviruses associated with CNS disease in literature and subsequently–given that the classical causality indicators are unfit for orthobunyaviral CNS disease–evaluated the strength of causality for each virus using tailor-made causality indicators. The results provide a comprehensible overview of orthobunyaviral CNS disease which can be used to guide future research.

## Introduction

The *Orthobunyavirus* genus comprises a wide range of arthropod-borne viruses which are prevalent worldwide and are commonly associated with severe disease in humans and other vertebrates [1]. One of the most severe types of orthobunyaviral disease are those of the central nervous system (CNS). A well-known example is La Crosse virus, the most common cause of paediatric encephalitis in the United States [2].

CNS disease associated with orthobunyaviruses can generally be classified into two types: congenital and postnatal. In humans, postnatal CNS disease is most commonly observed, such as meningitis and encephalitis [2,3], but only limited evidence exists for congenital disease [4]. In non-human vertebrates, on the other hand, congenital CNS disease is more common, such as hydranencephaly (absence of the cerebral hemispheres) in newborn ruminants [5], but postnatal disease is reported as well, often in the same species in which congenital disease is described [6]. In addition, some viruses are implicated in postnatal disease in one host (mostly humans) and in congenital disease in another host [7]. Combined, these data suggest that these two types of disease may be related.

A multitude of novel orthobunyaviruses have recently emerged [5,8] and several factors indicate that increasingly more will be discovered in the future. The latest report of the International Committee on Taxonomy of Viruses indicates that the number of recognized orthobunyavirus species has nearly doubled, from 49 in 2011 to 87 in 2019 [9]. This increase may be due to an actual increase in the rate of emergence, driven by factors such as mass animal farming, deforestation and climate change [10]. On the other hand, more viruses may currently be detected because the tools for detection have improved during the last decade [11,12].

With 87 recognized species and 214 named orthobunyaviruses known to date [9], there is a need to determine which viruses are relevant in a One Health context. The outbreak of the Schmallenberg virus in ruminants in Europe late 2011–causing foetal malformations and affecting more than 5,000 farms–demonstrated that emerging novel orthobunyaviruses can cause substantial problems in a short amount of time [13]. One of the key factors in determining which pathogens to focus on, is the ability to cause disease. Many orthobunyaviruses have been associated with CNS disease, however, evidence of viral infection does not implicate causality, and an overview of the causality evidence for orthobunyaviral CNS disease is lacking.

Historically, causality is proven by fulfilling all the Henle-Koch postulates [14]. Many of these postulates cannot be applied to orthobunyaviral CNS disease (e.g. all infected individuals should develop the disease which does not allow asymptomatic infections), are unethical (e.g. require healthy individuals to be inoculated by potentially lethal pathogens) or lack the specificity to evaluate features specific for viral CNS infections (e.g. localizing a pathogen to the CNS would support causality). More recently, a multitude of different postulates or ‘causality indicators’ for infectious diseases have been proposed, but a consensus on a definitive subset is lacking [15]. Consequently, there is a need for disease-specific causality indicators which allows evaluation of the strength of causality on a continuous scale, rather than a dichotomous result (i.e. causal or not causal).

The aim of this study was to provide an overview of orthobunyaviral CNS disease in vertebrates and objectify the strength of causality for each virus. We screened literature reporting cases of CNS infections where orthobunyaviruses were implicated as a potential cause and developed tailor-made causality indicators to suit orthobunyaviral CNS disease. For each of the orthobunyaviruses, we subsequently collected additional metadata to score each of the causality indicators and evaluate the evidence for causality.

## Methods

### Orthobunyavirus literature search

A literature search was performed in PubMed using a combination of the search terms on orthobunyaviruses and CNS diseases. References of all sources were checked for additional eligible articles. In addition, several textbooks and reviews on orthobunyaviruses and arbovirology were consulted. Primary sources (articles describing cases of orthobunyaviral CNS infections) were preferred but if not available, secondary sources (e.g. reviews) were also included. Viruses were considered to be associated with CNS disease when mentioned as such in the primary source or when patients demonstrated clear symptoms (decreased consciousness, personality changes, seizures, focal neurologic deficits or meningism) suggesting CNS involvement; i.e. patients with only a headache were not included. Viruses that only gave CNS disease upon experimental intracerebral inoculation were not included as this does not mimic natural infection, which requires that a virus, among other features, has to enter and traverse the body and cross the blood-brain barrier, which many pathogens cannot.

### Causality indicators and virus-specific data retrieval on causality indicators

Previously described causality indicators [15] were reviewed for their fitness to evaluate the association between orthobunyaviruses and CNS disease. Minor modifications to the causality indicators were allowed to make them more specific to orthobunyaviral CNS infections. For each of these specific causality indicators, criteria were established to quantify to which extent they were met. Data on each of the causality indicators, on natural host range and geographic distribution was collected through virus-specific additional literature searches and from the Centers for Disease Control Arbovirus Catalogue (ArboCat). Causality was reviewed from a viral perspective, as such, results of different types of CNS disease (e.g. congenital and postnatal) in different host species (e.g. humans and other vertebrates) were combined for a single virus. When multiple results were available per causality indicator (e.g. when different species were experimentally infected), the result which provided the highest score was chosen. To facilitate interpretation, we finally combined all the causality indicators to a total causality score per virus, and grouped the scores into three categories of causality strength: ‘weak’, ‘moderate’ and ‘strong’.

## Results

### Primary literature review results

**Fig 1:**
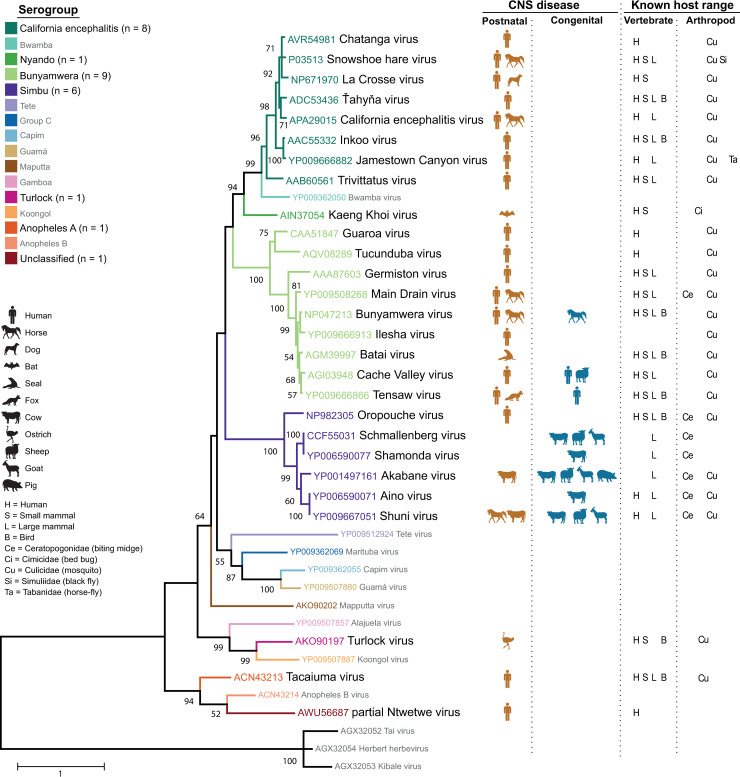
Phylogeny of 27 orthobunyaviruses linked to CNS disease in vertebrates. The tree is rooted by an outgroup of Herbeviruses (Tai virus, Herbert virus and Kibale virus), based on a recent phylogeny by Shchetinin *et al*. [64]. Nucleocapsid (S-segment) protein sequences were retrieved from reference phylogeny, and if not available, from GenBank (preferably RefSeq or Swiss-Prot). Protein sequences were aligned using M-Coffee and only columns with scores of 5 or higher were retained. A Maximum-Likelihood model with LG + G + I, complete deletion and a bootstrap of 1000 was used to infer the phylogeny in Mega v10. The phylogeny contains at least one virus from each of the known orthobunyavirus serogroups, in grey are representative viruses from serogroups without any virus linked to CNS disease. Between brackets: number of viruses associated with CNS infection in each specific serogroup. Coloured branches correspond to specific serogroups. Only Maximum Likelihood bootstrap values >50% are displayed. Pictograms indicate hosts associated with CNS disease, orange: postnatal disease, blue: congenital disease. Abbreviations describe the known host range (not per se requiring association with CNS disease), obtained from ArboCat [65] and other sources [30,66–68].

Of the 214 named orthobunyaviruses that are currently recognized [9], we identified 27 linked to CNS disease in vertebrates (**Table 1**). Seven viruses were linked to disease in humans and other vertebrates, 12 in humans alone and eight in non-human vertebrates alone. Non-human vertebrates with CNS disease included ruminants (cows, goats and sheep), horses, canines (dogs and foxes), bats, seals and ostriches. For humans, postnatal CNS disease was observed for all human-associated viruses, and congenital disease (together with postnatal CNS disease) only for Cache Valley virus and Tensaw virus. For non-human vertebrates, postnatal disease (11 of 15) and congenital disease (8 of 15) distributed nearly equally. The 27 orthobunyaviruses were found around the world, yet each virus had their specific geographic distribution, dictated by their vertebrate and arthropod host ranges. Phylogenetic analysis revealed that the viruses associated with CNS infections distributed across the known diversity of *Orthobunyavirus* serogroups and did not cluster in a monophyletic clade (**Fig 1**).

**Table 1:** Orthobunyaviruses associated with CNS infections in humans and other vertebrates.

		Human CNS disease					Non-human vertebrate CNS disease					Ref
Sero	Virus		Postnatal		Congenital			Postnatal		Congenital		
		Risk group	Description*	N	Disease	N	Host	Description*	N	Disease	N	
Anop A	TCMV	Adults	Deep coma	1								[16,17]
Bunyamwera	BATV						Seals	ME	1			[18]
	BUNV	Adults	Enc	2			Horses	Unspecified CNS disease	2	Abortion^†^	1	[17,19,20]
	CVV	Adults	Enc, acute/chronic men	4	Macro	2	Sheep			HE	5 outbreaks	[4,7,21–25]
	GERV	Adults	Mental confusion	1								[26]
	GROV	Adults	Prostration, paresis	4								[27,28]
	ILEV	Adults	ME	1								[29]
	MDV	Adults	Unspecified CNS disease	1			Horses	EM	6			[30,31]
	TENV	Adults	Enc	1	Micro, macro	0^‡^	Foxes	Rabies like symptoms	1			[4,32,33]
	TUCV	Children	ME	1								[34]
California	CEV	Children, adults	Enc	4^§^			Horses	Enc	2			[35,36]
	CHATV	Adults	Disorientation, nuchal rigidity	2								[37]
	INKV	Children, adults	Enc, men, polyradiculoneuritis	32								[37–39]
	JCV	Mostly adults	Men, ME	190^¶^								[40,41]
	LACV	Mostly children	Enc, men, ME	1,045^#^			Dogs	Men, necrotizing panenc	3			[40,42–44]
	SSHV	Mostly children	Enc, ME	8**			Horses	Enceph.	2			[45–47]
	TAHV	Children, adults	Enc, men, ME, chronic^††^	8								[38,39]
	TVTV	Adults	Paralysis	1								[48]
Nyando	KKV						Bats	Unspecified CNS disease	12			[49]
Simbu	AINOV						Cows			HE	Few outbreaks	[50,51]
	AKAV						Cows, sheep, goats, pigs	EM (cows)	Regular outbreaks	HE (all)	42,000^‡‡^	[6,52–54]
	OROV	Adults	Enc, men	>25								[55]
	SBV						Cows, sheep, goats			HE	>5,000 farms^§§^	[5,13,56,57]
	SHAV						Cows			HE	15	[58,59]
	SHUV						Cows, horses, sheep, goats	Enc, ME (horses, cows)	7 (5 horses, 2 cows)	HE (ruminants)	Few outbreaks	[60,61]
Turlock	TURV^¶^						Ostriches	EM	1			[62]
Unc	NTWV	Children	Enc	1								[8]

Ref, references; Sero, serogroup; Anop A, Anopheles A; Unc, Uncategorized; N, number of cases of CNS disease with acute infection described in literature unless otherwise specified; CEV, California encephalitis virus; CHATV, chatanga virus; GROV, Guaroa virus; INKV, Inkoo virus; JCV, Jamestown Canyon virus; LACV, La Crosse virus; SSHV, snowshoe hare virus; TAHV, Ťahyňa virus; TVTV, trivitattus virus; AINOV, Aino virus; AKAV, Akabane virus; OROV, Oropouche virus; SBV, Schmallenberg virus; SHAV, Shamonda virus; SHUV, Shuni virus; BATV, Batai virus; BUNV, Bunyamwera virus; CVV, Cache Valley virus; GERV, Germiston virus; ILEV, Ilesha virus; MDV, Main Drain virus; KKV, Kaeng Khoi virus; TENV, Tensaw; TCMV, Tacaiuma virus; TUCV, Tucunduba virus; TURV, Turlock virus; NTWV, Ntwetwe virus; Enc, encephalitis; Men, meningitis; ME, meningoencephalitis; EM, encephalomyelitis; Macro, macrocephaly; Micro, microcephaly; HE, hydranencephaly. Blue: associated with human CNS disease only, red: associated with non-human CNS disease only and green: associated with human and non-human vertebrate CNS disease.

*As described by the author.

^†^Virus detected in the brain of the aborted horse.

^‡^Disease association based on a significantly higher prevalence of antibodies (any type) in the mother of micro- and macrocephalic newborns than healthy newborns, however, no cases of acute infection described.

^§^504 cases described by one source[32], these were not included as they were likely associated with another virus from the same serogroup.

^¶^Cases reported in the US between from 2004 to 2018.

^#^Cases reported in the US between 2004 and 2018.

**Cases described in Canada.

^††^Also associated with several chronic neurologic infections[63]

^‡‡^Number of abnormal calves born between 1972 and 1975 in Japan.

^§§^ Number of farms reporting cases of SBV-associated congenital disease from December 2011 until May 2012.

^¶¶^The virus isolated from the ostrich was a Turlock-like virus, for this review considered as Turlock virus itself.

### Causality indicator selection

Twenty-five previously described causality indicators [15] were reviewed on their fitness to assess the causality of orthobunyaviral CNS infections in vertebrates (**Table 2**). Based on these indicators, six orthobunyaviral CNS disease indicators were developed as described below. For each of these six causality indicators, criteria were established to score to which extent each indicator was satisfied (range 0 to 3, **Table 3**).

**Table 2:** Classical causality indicators and fitness for orthobunyaviral CNS disease.

Causality indicator	Fit	Reason for unfitness or modified causality indicator (CI)
Parasite occurs in every case of the disease under circumstances that could account for observed pathology.	M	Higher prevalence in cases than controls (CI 1)
Parasite is absent from those without the disease.	M	Higher prevalence in cases than controls (CI 1)
It can be reproducibly grown in pure culture.	F	Could the virus be isolated? (CI 2)
It can induce the disease anew.	M	Also allowing experimental infection of other species (CI 3)
Specific virus must regularly be found associated with a disease.	U	Orthobunyaviruses often cause asymptomatic infection
Virus must be shown to occur in the sick individuals but not as an incidental or accidental finding, instead being the cause of the disease under investigation.	U	Orthobunyaviruses often cause asymptomatic infection
New virus established by laboratory passage (animal/tissue culture).	F	Could the virus be isolated? (CI 2)
Repeatedly isolated from human specimen and not a contaminant derived from host used to propagate the virus.	U	Unfavourable for rare or novel viruses
Antibody response increasing as a result of infection.	F	Included as diagnosis by serological criteria (CI 2)
Agent compared with other similar viruses.	F	Additional information congruent with biological knowledge (CI 6)
Constant association with specific illness.	U	Orthobunyaviruses often cause asymptomatic infection
Double blind studies with human volunteers should reproduce clinical disease.	M	Also allowing experimental infection of other species (CI 3)
Cross-sectional and longitudinal studies to identify patterns of disease.	F	Use of syndrome-based studies not limited to infected persons (CI 1)
Preventable by use of specific vaccine.	U	No vaccine available
Nucleic acid sequence belongs to a putative pathogen and is present in most cases of an infectious disease preferentially associated with pathology.	M	Higher prevalence in cases than controls (CI 1)
Lower copy number or absence of these sequences from those without disease.	M	Higher prevalence in cases than controls (CI 1)
Decrease or absence following treatment/recovery.	U	No treatment available
Detection predates disease or sequence copy number correlated with severity.	U	Asymptomatic orthobunyaviral infection can have significant viraemia
Congruence with biological knowledge.	F	Was additional information in line with a viral CNS infection? (CI 6)
Correlation with areas of tissue pathology.	F	Was additional information in line with a viral CNS infection? (CI 6)
Reproducible findings.	U	Unfavourable for rare or novel viruses
Sequencing microbial community.	M	Metagenomic sequencing to exclude other pathogens (CI 5)
Computational models to assess presence and proportion for resulting pathology.	U	Not applicable to orthobunyaviruses
Isolation of microbes of interest from diseased host.	F	Could the virus be isolated? (CI 2)
Testing of fresh isolates and consortia in relevant disease model.	F	Did experimental infection lead to similar symptoms (CI 3)

Adapted from Antonelli et al. [15]. M, modified; F, fit; U, unfit. The number in bracket describes for which orthobunyaviral CNS disease specific CI (Table 3) this classical CI was used.

**Table 3:** Orthobunyaviral CNS disease specific causality indicators.

1. What was the prevalence of acute infection in a cohort of clinically defined CNS infections?*	
a. ≥1/10% of cases had evidence of infection, no controls tested b. ≥5/50% of cases had evidence of infection, no controls tested c. Significantly higher in cases than controls	1 point 2 points 3 points
2. How was the infection established?	
a. By serological evidence of acute infection b. Same as a. and excluding cross-reactivity of related viruses^†^ c. By virus isolation or genome detection (PCR/sequencing)	1 point 2 points 3 points
3. What was the result of experimental infection? ^‡^	
a. CNS disease/death after inoculation in non-suckling rodents b. CNS disease/death after inoculation other species in same order c. CNS disease/death after inoculation in studied species	1 points 2 points 3 points
4. Where was the virus identified?	
a. Blood or other non-CNS material b. Cerebrospinal fluid or brain tissue c. Virus observed in in CNS cells	1 point 2 points 3 points
5. Were alternative causes for a CNS infection screened for and excluded?	
a. Excluded other likely viral aetiologies b. Excluded two pathogen types (e.g. viruses and bacteria) c. Excluded more than two pathogen types	1 point 2 points 3 points
6. Was additional (e.g. histopathological, immunological or imaging) information obtained and congruent with the current biological knowledge on viral CNS infections?	
a. Electro-encephalogram (EEG) b. CT, MRI or immunological markers c. Histopathology	1 point 2 points 3 points

* Infection established outside the CNS was scored one point lower. For a. and b., the higher prevalence is for livestock, the lower prevalence is for all other vertebrates. Only scored positive if at least 2 cases were detected.

† By also testing for other neurotropic viruses from the same serogroup known to circulate within the studied region.

‡ Disease after intracerebral inoculation was scored one point lower.

The first causality indicator compares the prevalence of acute infection in cases and healthy controls. Knowing that orthobunyaviruses can cause asymptomatic infections and that many other pathogens can also cause CNS infections, this indicator does not require the virus to be absent in controls, nor present in all cases. Instead, a significantly higher prevalence in cases than controls is considered as the strongest evidence (3 points). For cohorts without a control group, we determine that a certain prevalence, although suboptimal, can still support causality when a virus was localized to the CNS (see Discussion). Given that outbreaks in livestock are more likely to result in a higher prevalence because of their shared risk profile and close proximity, we consider that the threshold for prevalence in livestock should be higher to reach the same score. For livestock, the prevalence thresholds are >10% (1 point) and >50% (2 points). For other vertebrates, the thresholds are >1% (1 point) and >5% (2 points), with at least 2 positive cases.

Diagnostic methods for virus identification are assessed by the second indicator. We regard serological identification to be a weaker method (1 point) as it leaves the possibility for cross-reactivity. Although cross-reactivity cannot be completely excluded (see Discussion), attempts to eliminate cross-reactivity by including other viruses within the same serogroup is superior (2 points). To exclude previous infections, all serologically identified infections had to be confirmed as being acute (to confirm a temporal association). Due to heterogeneity in criteria for an acute infection, the criteria as described in the original reference are followed. Genome detection or virus isolation is considered superior and is therefore given 3 points.

The third indicator evaluates the capability of inducing similar disease after experimental infection. This indicator is fully satisfied (3 points) when CNS disease is observed after inoculation of the same host species as the index case. When inoculated in a different species within the same taxonomic order, two points are scored. The lowest (1 point) score is awarded when disease develops in experimentally infected rodents. No points are given when CNS disease could only be elicited in suckling mice (in contrast to weanling and adult mice) as they are well-known amplifiers of any *Bunyavirales* species [69]. A fatal outcome is regarded equal to developing CNS disease because severe CNS infections often result in death and post-mortem studies to prove CNS pathology may not always be performed. Besides outcome, this indicator also considers the route of inoculation. An orthobunyavirus inoculated outside the CNS virus needs to cross the blood-brain barrier whereas an intracerebrally inoculated virus does not. Similarly, for congenital CNS infections, a virus inoculated in pregnant mothers needs to cross the placenta in contrast to intrauterine inoculated viruses. Following this reasoning, intracerebrally or intrauterine inoculated viruses are scored one point lower.

The localization of the virus, the fourth causality indicator, is another important feature of causality. The highest score is awarded for viruses visualized intracellularly in the CNS (3 points) or when detected from brain biopsies. Two points are given when viruses are detected in CSF, which is scored lower because a virus may have ‘leaked’ into the CSF because of a disrupted blood-brain barrier, carried by migrating lymphocytes (e.g. Oropouche virus is known to infect leukocytes [70]) or because the CSF may have been contaminated with virus-containing blood during lumbar puncture. Viruses detected outside the CNS are given the lowest score (1 point).

The fifth causality indicator determines the exclusion of alternative aetiologies. Although this criterion was not specifically described in the original list, we consider this to be of importance as CNS disease can be caused by many pathogens. We regard the exclusion of three or more pathogen types (viruses, bacteria, fungi, parasites and prions) as the highest score (3 points), two pathogen types one point lower, and only one type of pathogen the lowest score (1 point). If a more likely alternative aetiology is identified, no points are awarded. When histopathological evidence suggests a viral cause and multiple alternative viral aetiologies are excluded, three points are awarded.

Causality indicator six evaluates whether additional evidence, congruent with the biological knowledge, is available to support an orthobunyaviral CNS infections. Brain tissue showing pathological changes indicative of a viral infection is considered as the strongest evidence (3 points). Brain imaging or immune profiling is also considered but regarded as less specific and therefore scored lower (2 points). EEG pattern indicative of a focal encephalopathy results in one point.

### Causality evaluation

Using the above mentioned causality indicators, we scored the 27 orthobunyaviruses associated with CNS infections in vertebrates (**Table 4**). The total causality scores ranged from 3 to the maximum of 18 points and were further narrowed into three categories: ‘weak’ (0–6 points), ‘moderate’ (7–12 points) and ‘strong’ (13–18 points). Akabane virus was the only virus that scored full points. Besides Akabane virus, La Crosse, Aino, Bunyamwera, Cache Valley, Schmallenberg, Shuni, Turlock-like and Jamestown Canyon virus all fell in the ‘strong’ causality category. Batai, Oropouche, Shamonda, Ntwetwe, Snowshoe Hare, Kaeng Khoi, Main Drain and Inkoo virus were categorized as ‘moderate’, and Ilesha, Ťahyňa, California encephalitis, Tensaw, Chatanga, Tacaiuma, Guaroa, Tucunduba, Germiston and Trivitattus virus had a ‘weak’ evidence of causality.

**Table 4:** Causality scores for orthobunyavirus associated with CNS infections in vertebrates.

Virus	1. Prevalence of acute infection in symptomatic versus control cohort (number of total cases studied)		2. Virus detection determined by		3. Experimental infection producing disease in		4. Virus localized in		5. Which other pathogen types were excluded		6. Additional evidence supporting viral CNS infection		Total score		Ref
AKAV	3	88% (59) vs 27% (11)*	3	Isolation	3	Pregnant cows IV	3	CNS cells	3	Viruses^†^	3	Histopathology	18	Strong	[6,53,71,72]
LACV	1	8–30%^‡^, no controls, ex CNS	3	Isolation	3	Puppies IV	3	CNS cells	3	>2 pathogen types	3	Histopathology	16		[2,42,73–75]
AINOV	0	10% (30), no controls, ex CNS	3	Isolation	3	Pregnant cows IV	3	CNS cells	3	Viruses^†^	3	Histopathology	15		[50,76–78]
BUNV	0	NR	3	Isolation	3	Humans IV^¶^	3	Brain tissue	3	>2 pathogen types	3	Histopathology	15		[20,79]
CVV	0	NR	3	Isolation	2	Pregnant sheep IU	3	CNS cells	3	>2 pathogen types^§^	3	Histopathology	14		[21,22,80]
SBV	1	41% (54), no controls, in CNS	3	Isolation	1	Adult mice SC	3	CNS cells	3	>2 pathogen types^§^	3	Histopathology	14		[5,56,81–83]
SHUV	2	100% (15), no controls, in CNS	3	Isolation	0	Newborn mice IP	3	Brain tissue	3	>2 pathogen types	3	Histopathology	14		[59,60,65]
TURV	0	5% (20), no controls, in CNS	3	Isolation	1	Weanling mice IP	3	Brain tissue	3	>2 pathogen types	3	Histopathology	13		[62,65]
JCV	0	NR	3	PCR	1	Weanling mice IP	3	Brain tissue	3	>2 pathogen types	3	Histopathology	13		[65,84]
BATV	0	NR	3	Isolation	0	Weanling mice IC	3	CNS cells	3	Viruses^†^	3	Histopathology	12	Moderate	[18,65]
OROV	1	3% (110), no controls, in CNS	3	PCR	1	Adult hamster SC	2	CSF	3	>2 pathogen types	2	CT	12		[85–87]
SHAV	0	NR	3	Isolation	0	Newborn mice IP	3	Brain tissue	3	Viruses^†^	3	Histopathology	12		[58,65]
NTWV	0	NR	3	PCR	0	NR	2	CSF	3	>2 pathogen types^§^	2	Immune profiling	10		[8]
SSHV	0	NR	2	Serology**	1	Weanling mice IP	1	Outside CNS	3	>2 pathogen types	3	Histopathology	10		[47,65,88]
KKV	2	92% (12) vs 0% (1), in CNS^#^	3	Isolation	0	Newborn mice IP	3	Brain tissue	1	Lyssavirus	0		9		[49,65]
MDV	0	NR	3	Isolation	1	Pregnant sheep IU^††^	3	Brain tissue	1	Viruses	0		8		[31,89]
INKV	1	20% (10), no controls, ex CNS	2	Serology**	0	Newborn mice IP	1	Outside CNS	2	Viruses and bacteria	1	EEG	7		[37,65,90]
ILEV	0	NR	3	Isolation	0	Newborn mice IC	2	CSF	1	Viruses	0		6	Weak	[29,65]
TAHV	0	10% (10), no controls, ex CNS^§§^	2	Serology**	1	Weanling mice IP	1	Outside CNS	2	Viruses and bacteria	0		6		[38,65,90]
CEV	0	1% (188), no controls, ex CNS	2	Serology**	1	Monkeys IC	1	Outside CNS	1	Viruses	1	EEG	6		[35,36,91,92]
TENV	0	0% (1,739)^‡‡^, no controls	3	Isolation	0	Newborn mice IP	3	Brain tissue	0	NR	0		6		[32,33,65]
CHATV	0	0% (4,214)^¶¶^, no controls, ex CNS	2	Serology**	0	NR	1	Outside CNS	2	Viruses and bacteria	0		5		[37]
TCMV	0	NR	3	Isolation	0	Newborn mice IP	1	Outside CNS	0	NR, but malaria positive	0		4		[16,65]
GROV	0	NR	3	Isolation	0	Newborn mice SC	1	Outside CNS	0	NR, but malaria positive	0		4		[16,65]
TUCV	0	NR	3	Isolation	0	NR	1	Outside CNS	0	NR	0		4		[28]
GERV	0	NR	2	Serology**	1	Weanling mice IP	1	Outside CNS	0	NR	0		4		[26,65]
TVTV	0	NR	2	Serology^##^	0	Newborn mice IP	1	Outside CNS	0	NR	0		3		[48,65]

Ref, references; IV, intravenous; IU, intrauterine; IP, intraperitoneal; SC, subcutaneous; IC, intracerebral.

*Significant difference, p = 0.0034, Fisher’s exact test.

^†^ Viral cause deemed most likely by histopathology.

^‡^Of all encephalitis cases in USA in 1999.

^§^Deep sequencing was performed to agnostically screen for different pathogen types.

^¶^Humans with inoperable neoplasms were inoculated to study potential oncolytic effects of this virus.

^#^Non-significant difference, p = 0.1538, Fisher’s exact test, but prevalence in cases >50% and detected in CNS.

**Acute infection determined using IgM testing or increasing neutralization titres at convalescence.

^††^Intrauterine inoculation of pregnant sheep resulting in congenital CNS disease although MDV is only associated with postnatal CNS disease. However, because of the high co-occurrence of postnatal and congenital disease, this was deemed as relevant.

^‡‡^ 1 of 1739, unknown whether infection was determined by serology (ex CNS) or isolated from CSF or brain tissue (in CNS).

^§§^A significantly higher seroprevalence (p = 0.012) was found in patients with chronic disseminated encephalomyelitis (37%, 13 of 37) than in healthy controls (9%, 6 of 69), but was not determined to be an acute infection[63].

^¶¶^2 of 4214.

^##^Presumptive recent infection determined by various serologic criteria.

## Discussion

The large and increasing number of orthobunyaviruses associated with CNS disease makes it challenging to determine importance from a One Health perspective. A key feature of determining importance is causality, yet classical causality tests are unfit for orthobunyaviral CNS disease. We developed a list of tailored causality indicators, identified 27 orthobunyaviruses to be associated with CNS disease in vertebrates and evaluated their strength of causality. We identified several viruses with high causality scores which may be regarded as ‘causal’ and found numerous viruses with lower causality scores, for which we identify research gaps to focus on.

Eight orthobunyaviruses had a ‘strong’ causality score. These include Akabane, Aino, Shuni and Schmallenberg virus, commonly associated with congenital CNS malformations in ruminants. La Crosse, Jamestown Canyon, Cache Valley and Bunyamwera virus also belong to this group, which are mostly known for human postnatal disease. Akabane virus was the only virus able to completely satisfy all causality indicators. Indeed, for the other seven viruses, the lack of case-control studies is a limitation (discussed below), but also appropriate experimental infection studies were missing. Understandably, experimental infections may not have been performed because of ethical constraints–especially for humans–yet surprisingly, this has been done for Bunyamwera virus. One report describes the experimental infection of humans with inoperable neoplasms with Bunyamwera virus as a potential oncolytic therapy, morbidly resulting in a fatal encephalitis [79]. While performing such experiments may support causality, we evidently do not advocate this. Rather, we consider sufficient causality evidence is available for these viruses to consider them causal.

Viruses classified as having a ‘moderate’ causality score included some well- and lesser-known viruses associated with CNS disease in humans and other vertebrates. For most of these viruses no case-control studies were done, experimental infections were only successful in rodents, and almost no viruses were visualized intracellularly in the CNS. Interestingly, this group includes Shamonda virus, commonly described in bovine congenital CNS disease, but despite the high prevalence lacks causality evidence to be ‘strong’. We consider that these viruses with a ‘moderate’ score are possible causes of CNS infections. Nonetheless, this group also includes Snowshoe Hare and Inkoo virus, which have never been detected in the CNS and for which only serological evidence of infection exists, allowing misclassification because of cross-reactivity. Considering these limitations, we suggest that more research into causality should be done for this group of viruses.

The remaining ten viruses all scored poorly on most causality indicators resulting in a ‘weak’ causality score. They were all found in humans, although some were also mentioned in disease in other vertebrates. The majority of serologically diagnosed viruses fell into this category, which stresses the importance of direct virus detection (e.g. PCR or isolation), preferably on CNS samples to strengthen causality. Even though Ilesha and Tensaw virus were isolated from the CNS, they failed to perform well on all other causality indicators. An explanation for this could be that these viruses are relatively ‘old’–they were last implicated in CNS disease in 1964 [29], and 1965 [33] respectively–when several conventional assays which may support causality (e.g. PCR or in situ hybridization) were not available. Similarly, even for more recently detected viruses, some of these experiments may not yet have been performed. These low causality scores therefore do not exclude the possibility of causality.

Case-control studies, often referred to as ‘association-studies’, are a fundamental part of causality research. Only one proper case-control study has been done for the exposure to Akabane virus in diseased and healthy newborn calves. For cohorts of cases with CNS disease, the prevalence of a single cause is usually very low, as there is an extensive list of potential causes of CNS disease. In such cohorts of diseased cases, even a low prevalence can indicate causality, but may similarly be a result of unrelated (co-)infection as orthobunyaviruses may commonly cause asymptomatic infection. To account for this, comparing the prevalence to a cohort without the disease would strengthen causality. For example, 4,214 humans with a suspected CNS infection were tested for Chatanga virus of which 2 were positive by serological evidence of acute infection. Because no healthy control group was added, it remains difficult to evaluate a causal relationship. An exception could be made when the pathogen is detected in the CNS, which is generally considered a sterile site and presence of a pathogen is more likely to be symptomatic and causal. In addition, it has to be kept in mind that sampling of CNS material is unethical from healthy subjects, hindering case-control studies. On the other hand, obtaining serum samples from healthy matched controls is much easier. Therefore, for diagnostics using non-CNS derived samples like blood, we strongly recommend the use of properly designed case-control studies as a fundamental aspect of proving causality.

Detection of orthobunyaviruses is commonly based on serologic methods, mainly because of their wide availability and their extended window period compared to virus detection based methods. However, serological assays may significantly influence causality because of two potential problems: temporality and cross-reactivity. Most of the current evidence that orthobunyaviruses may cause congenital CNS malformations in humans is based on a study (on Cache Valley and Tensaw virus) describing a significantly higher seroprevalence in mothers of micro- and macrocephalic newborns than healthy controls [4]. Because IgG antibody levels were measured in this study, a temporal association between onset of infection and disease is lacking. Conversely, a study on human postnatal CNS disease and Jamestown canyon virus found a significantly lower seroprevalence of reactive antibodies in cases (8%) than controls (11%), which, using the same logic, could argue against causality [48]. For this reason, we only deemed serological diagnoses with confirmation of acute infections relevant for this manuscript.

A second issue with serological assays is the possibility of cross-reactivity. The orthobunyaviruses are commonly divided into serogroups of viruses with serological cross-reactivity. Whereas the specificity of serological assays has greatly increased over the last years, cross-reactivity can only be conclusively excluded when all other viruses within the same serogroup are tested for. Given that we identified nine viruses within the Bunyamwera serogroup to be associated with CNS infections (**Table 1** and **Fig 1**), all of these should be included. One could argue that some of these viruses do not circulate in a certain region, at a given time or in a specific host, yet this knowledge may not always be available. For example, it is suggested that Jamestown Canyon virus infections in the United States have commonly been misclassified as La Crosse virus infections because of cross-reactivity [41].

It is important to note that several factors, such as the use of serological assays, but also differences in epidemiology and availability of research funding, have likely influenced the availability of causality evidence and subsequently the causality scores. The results of this study should therefore not be used to exclude causality of viruses with moderate or low causality scores. Rather, our study shows which viruses can be considered causal and reveals the knowledge gaps for the other viruses with lower causality scores, targets for future research.

One possible reason that many orthobunyaviruses (and other pathogens) are understudied is because of the large and increasing number of pathogens associated with CNS disease known to date. Testing for all pathogens using conventional diagnostics is impossible, and therefore clinicians and researchers usually are limited to those with a high prevalence and ‘proven’ causality, further biasing the availability of causality evidence. This may, in part, even explain why the cause of CNS disease frequently remains unknown [93], if these understudied pathogens are responsible for disease but not tested for. One promising alternative virus detection method, which increases the diagnostic yield and removes bias against understudied viruses, is metagenomics. In theory, metagenomics allows for detection of all pathogens–including potentially novel pathogens–by agnostic sequencing of the genomic content in a sample [11,12].

The observation that CNS disease was associated to 8% of the known orthobunyaviruses (17 had a ‘moderate’ or ‘strong’ causality score of the 214 that are known) could indicate that only a few have the capability to cause CNS disease. On the other hand, these CNS disease-related viruses are not phylogenetically or serologically clustered (**Fig 1**), which may actually suggest that all orthobunyaviruses have an intrinsic ability to cause CNS disease. This is supported by studies which have shown that multiple viruses, including ones not implicated in natural CNS disease such as San Angelo virus, can replicate in brain tissue after intrauterine inoculation in sheep [89]. Furthermore, this ability may even be host-independent, as we observed ten viruses to be implicated in CNS disease in multiple hosts of which seven included humans.

Determining importance from a One-Health perspective is, next to causality, also determined by the burden of disease, which this review does not primarily address. We summarized the total number of cases of human CNS disease described in scientific literature (**Table 1**), but this is a large underrepresentation of the actual prevalence. Only the number of human La Crosse virus and Jamestown canyon virus infections described in literature may be informative, as they are based on proper epidemiological studies [3,40], yet similar studies were not available for the other orthobunyaviruses. In terms of severity of disease, human fatalities from CNS disease were described for Ilesha [29], Tucunduba [34], Ntwetwe [8], Jamestown Canyon [94] and La Crosse virus [95]. For the first three, these were the only reported cases of CNS disease, but for the latter two, fatalities were rare. With respect to human congenital disease, the burden of disease seems to be low, as abortions were not reported and malformations were only reported in two putative cases of neonatal macrocephaly for whom a maternal diagnostic antibody rise against Cache Valley virus was found during pregnancy [4].

For this review we used a ‘glass half-full’ approach, as we only regarded evidence which would support causality and, if multiple results were available, scored the results which would support causality best. Consequently, studies reporting negative results–arguing against causality–were not considered. This was done because a positive result generally proves ability (e.g. isolation proves viability), whereas a negative result does not necessarily exclude this (e.g. failure to culture a virus does not prove absence of a viable virus in the patient). For example, one report describes the inability to visualize Main Drain virus in the brains of horses from which the virus was repeatedly isolated despite extensive attempts [31]. This does not exclude viral CNS tropism as the virus may have left the brain during an earlier stage of disease. Clearly, including negative results into a causality model would strengthen its value but is arguably complicated, yet doing so would be a direction of future research.

According to our knowledge, this is the first complete overview of orthobunyaviruses associated with CNS disease and one of the first to systematically evaluate the causality of multiple pathogens associated to a specific disease. This comprehensible overview can be used to identify viruses which may be regarded as proven causal, reveal research gaps for viruses with low causality scores and provides a framework to evaluate the causality of orthobunyaviruses that may newly emerge.
